# *HSPB7* regulates osteogenic differentiation of human adipose derived stem cells via ERK signaling pathway

**DOI:** 10.1186/s13287-020-01965-4

**Published:** 2020-10-23

**Authors:** Chanyuan Jin, Ting Shuai, Zhihui Tang

**Affiliations:** Second Clinical Division, Peking University School and Hospital of Stomatology, Beijing, 100081 China

**Keywords:** Osteogenesis, Bone formation, Heat shock protein, cvHSP

## Abstract

**Background:**

Heat shock protein B7 (*HSPB7*), which belongs to small heat shock protein family, has been reported to be involved in diverse biological processes and diseases. However, whether *HSPB7* regulates osteogenic differentiation of human adipose derived stem cells (hASCs) remains unexplored.

**Methods:**

The expression level of *HSPB7* during the osteogenesis of hASCs was examined by quantitative reverse transcription polymerase chain reaction (qRT-PCR) and Western blot analysis. Lentivirus transfection was used to knock down or overexpress *HSPB7*, which enabled us to investigate the effect of *HSPB7* on osteogenic differentiation of hASCs. U0126 and extracellular signal-regulated protein kinase 1/2 (ERK1/2) siRNA were used to identify the mechanism of the *HSPB7*/ERK1/2 axis in regulating osteogenic differentiation of hASCs. Moreover, ectopic bone formation in nude mice and osteoporosis mice model was used to investigate the effect of *HSPB7* on osteogenesis in vivo*.*

**Results:**

In this study, we found the expression of *HSPB7* was significantly downregulated during the osteogenic differentiation of hASCs. *HSPB7* knockdown remarkably promoted osteogenic differentiation of hASCs, while *HSPB7* overexpression suppressed osteogenic differentiation of hASCs both in vitro and in vivo. Moreover, we discovered that the enhancing effect of *HSPB7* knockdown on osteogenic differentiation was related to the activation of extracellular signal-regulated protein kinase (ERK) signaling pathway. Inhibition of ERK signaling pathway with U0126 or silencing ERK1/2 effectively blocked the stimulation of osteogenic differentiation induced by *HSPB7* knockdown. Additionally, we found that *HSPB7* expression was markedly increased in mouse bone marrow mesenchymal stem cells (mBMSCs) from the osteoporotic mice which suggested that *HSPB7* might be utilized as a potential target in the development of effective therapeutic strategies to treat osteoporosis and other bone diseases.

**Conclusion:**

Taken together, these findings uncover a previously unrecognized function of *HSPB7* in regulating osteogenic differentiation of hASCs, partly via the ERK signaling pathway.

## Introduction

Human adipose derived stem cells (hASCs) are multipotential mesenchymal stem cells (MSCs) that have the ability to differentiate into different lineages such as chondrocytes, adipocytes, and osteocytes [1]. As a type of MSCs, hASCs offer new therapeutic solutions for bone defects and metabolic diseases due to their abundant sources, ease of acquisition, low immunogenicity, and osteogenic differentiation capacity [2, 3]. However, the underlying mechanisms by which hASCs undergo osteogenic differentiation are not yet fully elucidated, thereby hindering their potential clinical applications.

It is well known that osteogenic differentiation is orchestrated by a large number of signaling pathways, such as the Wnt/β-catenin signaling pathway, phosphatidylinositol-3-kinases (PI3K)/protein serine threonine kinase (AKT) signaling pathway, transforming growth factor β (TGFβ) /bone morphogenetic protein (BMP) signaling pathway, and mitogen-activated protein kinase (MAPK) signaling pathway [4–7]. Among them, the extracellular signal-regulated kinase (ERK)/MAPK signaling pathway plays important roles in driving the commitment of MSCs into osteogenic lineage [8]. Researchers have revealed that ERK/MAPK could strongly activate the osteogenic-related transcription regulators such as runt-related transcription factor 2 (*RUNX2*) and osterix (*OSX*) [9]. Inactivation of ERK/MAPK signaling pathway in osteoprogenitors results in severe osteopenia and cleidocranial dysplasia [10]. Fibroblast growth factor receptor 1 (*FGFR1*), a transmembrane receptor which transduces extracellular signals to multiple intracellular downstream pathways, is indispensable for skeletal development via regulating osteoblast growth and differentiation [11]. Accumulating evidence has demonstrated that *FGFR1* exerts its function mainly through the activation of ERK/MAPK signaling [12].

Heat shock protein B7 (*HSPB7*), also known as cardiovascular heat shock protein (cvHsp), belongs to the small heat shock protein family with essential roles in maintaining cellular homeostasis in a variety of both physiological and pathophysiological processes [13–15]. *HSPB7* is highly expressed in the cardiac tissue and plays a critical role in cardiac metabolism [16]. The dysfunction of *HSPB7* is strongly associated with heart failure, dilated cardiomyopathy, and idiopathic cardiomyopathy [17]. *HSPB7* has also been demonstrated to function as a tumor suppressor in multiple malignancies such as renal cell carcinoma [18, 19]. However, the effects of *HSPB7* on osteogenic differentiation still remain unclear.

In this study, we evaluated the function of *HSPB7* in the osteogenic differentiation of hASCs both in vitro and in vivo. We found *HSPB7* knockdown promoted osteogenic differentiation of hASCs via activation of the ERK signaling pathway. These findings could contribute to better understanding of the molecular mechanisms underlying osteogenic differentiation and provide new therapeutic target for treating bone diseases.

## Material and methods

### Cell culture

Primary human adipose derived stem cells (hASCs), human bone marrow mesenchymal stem cells (hBMSCs), and mouse MC3T3-E1 cells were purchased from ScienCell (Carlsbad, CA, USA). Human osteosarcoma U2OS cells were obtained from China Infrastructure of Cell Line Resource (Beijing, China). Cells were cultured using a proliferation medium (PM) consisting of Dulbecco’s modified Eagle’s medium (DMEM) (Gibco, Grand Island, NY, USA), 10% fetal bovine serum (Gibco), and 1% penicillin/streptomycin (Gibco). For osteogenic differentiation, hASCs and hBMSCs were cultured in osteogenic medium (OM) containing standard PM supplemented with 0.2 mM ascorbic acid (Sigma, St. Louis, MO, USA), 10 mM β-glycerophosphate (Sigma), and 100 nM dexamethasone (Sigma). All experiments were repeated at least three times.

### Lentiviral transfection

In order to overexpress *HSPB7*, the lentivirus expressing *HSPB7* and the scramble negative control (NC) were purchased from Cyagen Company (Guangzhou, China). For *HSPB7* knockdown, recombinant lentiviruses targeting *HSPB7* (sh*HSPB7*-1 and sh*HSPB7*-2) and the non-targeting negative control (shNC) were purchased from GenePharma Co. (Shanghai, China). The shRNA sequences were as follows: shNC, TTCTCCGAACGTGTCACGT; sh*HSPB7*-1, ACAGAACCUCUUCCACCUUTT; and sh*HSPB7*-2, GAACACCUUCGCUCACAAGTT. For virus transfection, cells were exposed to the lentiviral supernatant with the addition of polybrene (5 μg/mL, Sigma-Aldrich) for 24 h. After 72 h, antibiotic selection was conducted by adding puromycin (5 μg/mL, Sigma-Aldrich) to transfected cells.

### Small interfering RNA

Small interfering RNAs (siRNAs) targeting ERK1/2 (siERK1, siERK2) and the negative control siRNA (siNC) were purchased from Integrated Biotech Solutions Co. (Ibsbio Co., Shanghai, China). The sequences were as follows: siNC, UUCUCCGAACGUGUCACGUTT; siERK1, GACCGGAUGUUAACCUUUAUU; and siERK2, CAGGGUUCCUGACAGAAUAUU. To knock down ERK1/2, cells were transfected with a mixture of siERK1 and siERK2 using the Lipofectamine 3000 transfection reagent (Invitrogen, Carlsbad, CA, USA) following the manufacturer’s instructions.

### Alkaline phosphatase (ALP) staining

Cells were cultured in PM or OM for 7 days. After that, cells were washed with PBS, fixed in 4% paraformaldehyde for 15 min, incubated with the ALP substrate solution (CoWin Biotech, Beijing, China), and recorded by a scanner (Image Scanner III, GE Healthcare Bio-Sciences Corp., Piscataway, NJ, USA).

### Quantification of ALP activity

Cells were washed with PBS, treated with 1% Triton X-100 (Sigma), and assayed with the ALP Activity Kit (Biovision, Milpitas, CA). ALP activity was quantified by reading the absorbance at 520 nm and normalized to the total protein content. Total protein concentration was determined using a BCA protein assay kit (Pierce Thermo Scientific, Waltham, MA, USA).

### Alizarin Red S (ARS) staining and quantification

At 14 days after osteogenic induction, cells were washed three times with PBS, fixed with 4% paraformaldehyde for 15 min, and rinsed with Milli-Q water. Next, the calcium deposition was stained with 1% Alizarin Red S solution (pH 4.2, Sigma) for 20 min. For quantification, the stain was dissolved in cetylpyridinium chloride (Sigma) and measured with a spectrophotometer at 562 nm.

### Oil Red O staining

To induce adipocyte differentiation, hASCs were cultured in adipocyte differentiation medium (AM) consisting of standard PM supplemented with 50 nM insulin (Sigma), 0.5 mM 3-isobutyl-1-methylxanthine (Sigma), 100 nM dexamethasone (Sigma), and 200 mM indomethacin (Sigma). At day 14 after adipogenic induction, cells were washed twice with PBS, fixed with 10% formalin for 30 min, and stained with Oil Red O working solution (0.3%, Sigma). Then, the stained cells were washed with distilled water and recorded by a microscope.

### Immunofluorescence staining

Cells grown on the glass coverslips were washed with ice-cold PBS, fixed with 4% formaldehyde, permeabilized with 0.2% Triton X-100, and then blocked with 1% bovine serum albumin. Next, cells were incubated with primary antibody against OCN (Abcam) at 4 °C overnight and then incubated with a fluorescence conjugated secondary antibody for 60 min at room temperature. After washing with PBS, cells were stained with DAPI for detection of nuclei. Images were visualized using a confocal microscope (Carl Zeiss, Oberkochen, Germany).

### RNA isolation and quantitative reverse transcription polymerase chain reaction (qRT-PCR) analysis

Total RNA was extracted using the TRIzol Reagent (Invitrogen) following the manufacturer’s instructions. The reverse transcription was performed with the PrimeScript RT Reagent Kit (Takara, Tokyo, Japan). qRT-PCR was implemented with SYBR Green Master Mix (Roche Applied Science, Mannheim, Germany) on a 7500 Real-Time PCR Detection system (Applied Biosystems, Foster City, CA, USA) with gene-specific primers. The relative expression levels were measured by comparative cycle threshold (CT) method.

The primer sequences used are as follows: *HSPB7*, forward (F) ACTTCTCACCTGAAGACATCATTG, reverse (R) CATGACAGTGCCGTCAGC; glyceraldehyde 3-phosphatedehydrogenase (*GAPDH*, internal control), (F) GGTCACCAGGGCTGCTTTTA, (R) GGATCTCGCTCCTGGAAGATG; *ALP*, (F) ATGGGATGGGTGTCTCCACA, (R) CCACGAAGGGGAACTTGTC; *RUNX2*, (F) CCGCCTCAGTGATTTAGGGC, (R) GGGTCTGTAATCTGACTCTGTCC; osteocalcin (*OCN*), (F) CACTCCTCGCCCTATTGGC, (R) CCCTCCTGCTTGGACACAAAG; *FGFR1*, (F) CTCATCTCCTGCATGGTGGG, (R) CTGGAGTCAGCAGACACTGTT; *PPARγ*, (F) GAGGAGCCTAAGGTAAGGAG, (R)GTCATTTCGTTAAAGGCTGA; *BMPRIA*, (F) TTCCCTGGGGTCCGGACTTA, (R) CTGCTTTCTTACGACTCCTCCA; *BMPRIB*, (F) TCTATGCACACAAGGGCAAAC, (R) TGGTGGTGGCATTTACAACG; *BMPRII*, (F) TGCAGCCATAAGCGAGGTTG, (R) CCCTCAAGTTCACAGCTCCT; *IGF1R*, (F) ACGAGTGGAGAAATCTGCGG, (R) ATGTGGAGGTAGCCCTCGAT; mouse *Gapdh*, (F) GGGTTCCTATAAATACGGACTGC, (R) TACGGCCAAATCCGTTCACA; and mouse *Hspb7*, (F) CGGGCTGAGAAGCTGGCA, (R) GTTGGGTCCACATCCTCTGG.

### Protein extraction and Western blot analysis

Total cellular protein extracts were obtained with radioimmunoprecipitation assay (RIPA) lysis buffer supplemented with 1% PMSF (Sigma) and 1% phosphatase inhibitor (Roche Applied Science). The concentration of protein was measured by the BCA protein assay kit (Thermo). Next, equal amounts of samples were separated by 10% sodium dodecyl sulfate polyacrylamide gel electrophoresis and transferred to polyvinylidene fluoride (PVDF) membranes (Millipore, Billerica, MA, USA). After being blocked with skimmed milk in TBST buffer (0.1 M Tris, 150 mM NaCl, and 0.1% Tween-20), the membranes were incubated overnight at 4 °C with primary antibodies against HSPB7 (Abcam, Cambridge, UK), RUNX2 (Abcam), OCN (Abcam), ERK1/2 (Cell Signaling Technology, Beverly, MA, USA), phosphorylated-ERK1/2 (Thr202/Tyr204) (Cell Signaling Technology), and GAPDH (HuaxingBio Science, Beijing, China). Subsequently, the membranes were washed three times with TBST and incubated with horseradish peroxidase (HRP) conjugated secondary antibodies for 1 h at room temperature. The bands were visualized via the ECL Western Blot Kit (CoWin Biotech). The intensity of bands was quantified using ImageJ analysis software (http://rsb.info.nih.gov/ij/) and normalized to GAPDH.

### Ectopic bone formation in vivo

All animal protocols were performed in accordance with the laboratory animal care and use guidelines and approved by the Peking University Animal Care and Use Committee. Thirty BALB/c homozygous nude (nu/nu) male mice (8 weeks, 18–20 g) purchased from Vital Co. (Beijing, China) were randomly divided into five groups (*n* = 6 per group). Cells were induced in OM for 7 days, resuspended, incubated with Bio-Oss Collagen (Geistlich, GEWO GmbH, Baden-Baden, Germany) for 1 h at 37 °C, and then implanted into the dorsal subcutaneous sites of nude mice. After 8 weeks, the implants were harvested and fixed with 4% formalin for 24 h, decalcified in 10% EDTA for 14 days, and followed by embedding in paraffin. Sections (5 μm thick) were cut and stained with hematoxylin and eosin (HE) and Masson’s trichrome.

### Establishment of ovariectomized mouse model

Eight-week-old female BALB/c mice weighting 20 ± 2 g were obtained from Vital Co. After 1 week of acclimatization, the mice were randomly allocated to two groups: sham operation group (SHAM) and ovariectomized group (OVX). The ovariectomy was performed as described previously [20]. Twelve weeks following the ovariectomy surgery, femurs were collected and evaluated using a micro-computed tomography (micro-CT) system. The scanner was set at a current of 220 μA, a voltage of 60 kV, and a resolution of 9.088 μm per pixel. Inveon Research Workplace (Inveon, Siemens, Munich, Germany) was used to calculate morphometric parameters, including bone volume/total volume (BV/TV), trabecular separation (Tb.Sp), and trabecular number (Tb.N) in the trabecular region (1 to 2 mm distal to the proximal epiphysis). Then, the femoral specimens were decalcified in 10% EDTA solution, embedded in paraffin, and stained with hematoxylin and eosin (HE). mBMSCs were collected from the tibias of SHAM and OVX mice as described previously [21].

### Statistical analysis

All data were expressed as mean ± standard deviation (SD). The data were calculated with SPSS statistics version 16.0 (SPSS Inc., Chicago, IL, USA). Independent sample *t* test was used for comparison of two groups, and one-way analysis of variance (ANOVA) was applied for the comparison among multiple groups. A value of *P* < 0.05 was considered statistically significant.

## Results

### *HSPB7* was downregulated during osteogenic differentiation of hASCs

**Fig. 1:**
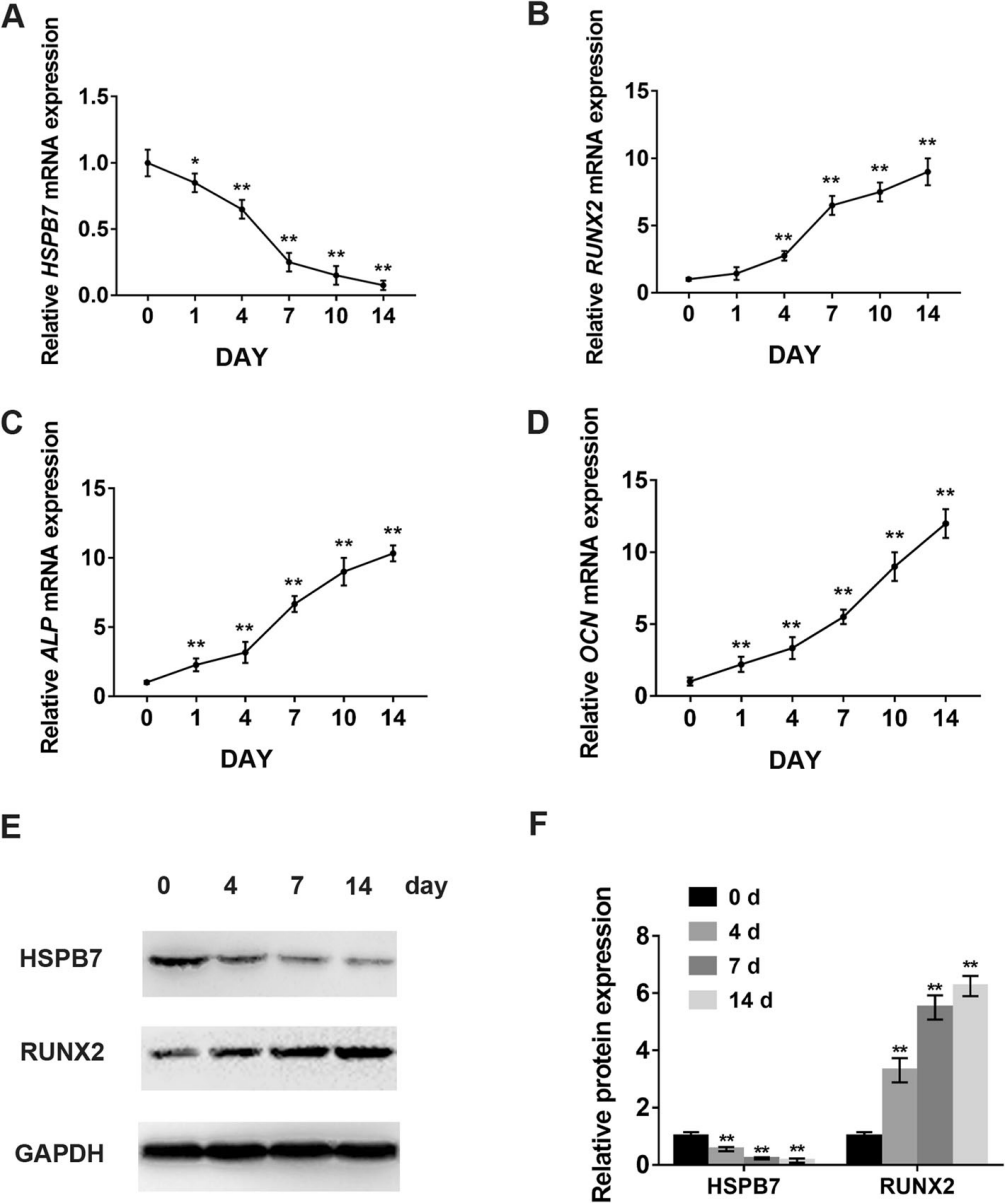
*HSPB7* was downregulated during the osteogenic differentiation of hASCs. **a** Relative mRNA expression of *HSPB7* was measured by qRT-PCR at days 0, 1, 4, 7, 10, and 14 during the osteogenic differentiation of hASCs. **b**–**d** Relative mRNA expression of *RUNX2*, *ALP*, and *OCN* during the osteogenic differentiation of hASCs. **e** After osteogenic induction, the protein expression of HSPB7 was significantly reduced, accompanied by the increased expression of RUNX2. **f** The quantitative results of **e** by Image J software. Glyceraldehyde 3-phosphate dehydrogenase (GAPDH) was used as a control. Data are presented as the mean ± SD (*P < 0.05, **P < 0.01, compared with day 0)

In order to determine the effect of *HSPB7* in the process of osteogenesis, we first detected the expression level of *HSPB7* during osteogenic differentiation of hASCs. qRT-PCR analysis showed the mRNA expression of *HSPB7* was remarkably decreased during osteogenesis, while the expression levels of osteogenic markers *RUNX2*, *ALP*, and *OCN* were significantly upregulated (Fig. 1a–d). Consistently, Western blot revealed a similar trend in protein expression (Fig. 1e, f).

Moreover, we compared the expression of *HSPB7* among hASCs, hBMSCs, and human osteoblast-like U2OS cells. Results showed that *HSPB7* was expressed at a much lower level in U2OS cells compared with hASCs and hBMSCs (SFig. 1A). Meanwhile, we compared the expression level of *Hspb7* between mBMSCs and mouse MC3T3-E1 cells and found that the level of *Hspb7* was very low in mouse MC3T3-E1 cells compared with mBMSCs (SFig. 1B). These results indicated that *HSPB7* might be associated with the stage of osteogenic differentiation. Additionally, we found long-term expansion of hASCs in vitro affected the expression of *HSPB7*. As shown in SFig. 1C, hASCs at late passages have a higher expression of *HSPB7* compared with hASCs at early passages.

### *HSPB7* overexpression inhibited osteogenic differentiation of hASCs

**Fig. 2:**
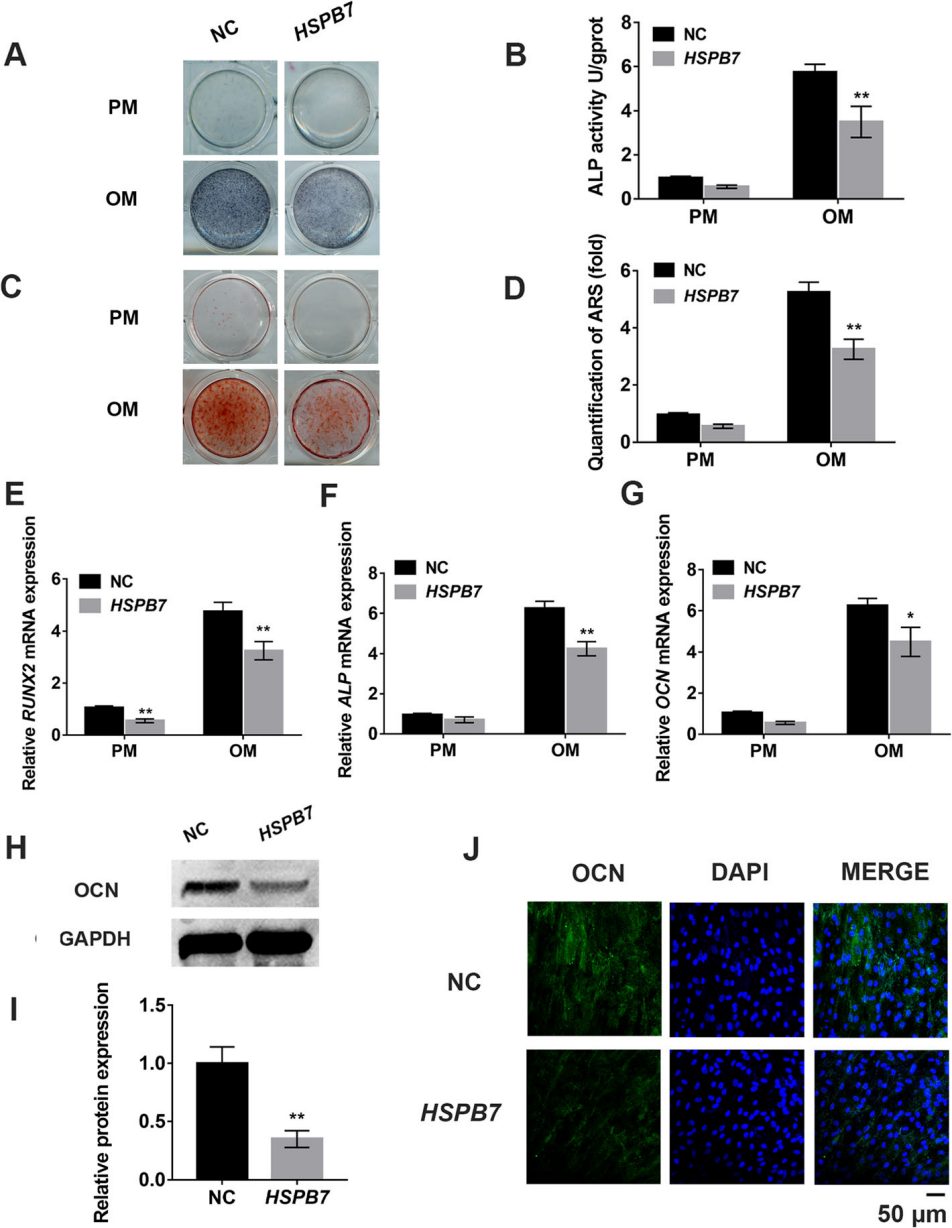
*HSPB7* overexpression inhibited the osteogenic differentiation of hASCs in vitro. **a** ALP staining in the *HSPB7* overexpression (*HSPB7*) and control (NC) groups at day 7 of osteogenic differentiation. **b** Quantification of ALP activity in *HSPB7* overexpression and control hASCs at day 7 of osteogenic differentiation. **c** Alizarin Red S staining was performed at day 14 of osteogenic differentiation. **d** Quantification of Alizarin Red S (ARS) staining. **e**–**g** Relative mRNA expression of osteogenic genes *RUNX2*, *ALP*, and *OCN* was assessed by qRT-PCR at day 14 of osteogenic differentiation. **h**, **i** *HSPB7* overexpression downregulated the protein expression of OCN, as determined by Western blot. **j** Confocal microscopy confirmed the downregulation of OCN protein expression in *HSPB7* overexpression hASCs at day 14 of osteogenic differentiation. Scale bar = 50 μm. Data are presented as the mean ± SD (*P < 0.05, **P < 0.01, compared with NC)

To clarify whether *HSPB7* regulates osteogenic differentiation of hASCs, we overexpressed *HSPB7* in hASCs by lentivirus transfection. Fluorescent staining revealed the transfection efficiency was more than 90% (SFig. 2A). Western blot and qRT-PCR showed the expression level of *HSPB7* increased by more than 15 folds in *HSPB7* overexpression hASCs in comparison to the negative control hASCs (NC) (SFig. 2B-D). After culturing the infected hASCs in OM for 7 days, ALP activity was inhibited in *HSPB7* overexpression cells compared to NC cells, as revealed by ALP staining and quantitative ALP activity assay (Fig. 2a, b). Similarly, extracellular matrix mineralization was also decreased in the *HSPB7* overexpression group, as determined by ARS staining and quantification on day 14 (Fig. 2c, d). Furthermore, qRT-PCR was performed to detect the mRNA expression levels of osteogenic markers, which showed that *HSPB7* overexpression downregulated the expression of *RUNX2*, *ALP*, and *OCN* after 14 days of osteogenic induction (Fig. 2e–g). Additionally, Western blot and immunofluorescence showed the protein expression of OCN was reduced in the *HSPB7* overexpression group (Fig. 2h–j).

### *HSPB7* knockdown enhanced osteogenic differentiation of hASCs

**Fig. 3:**
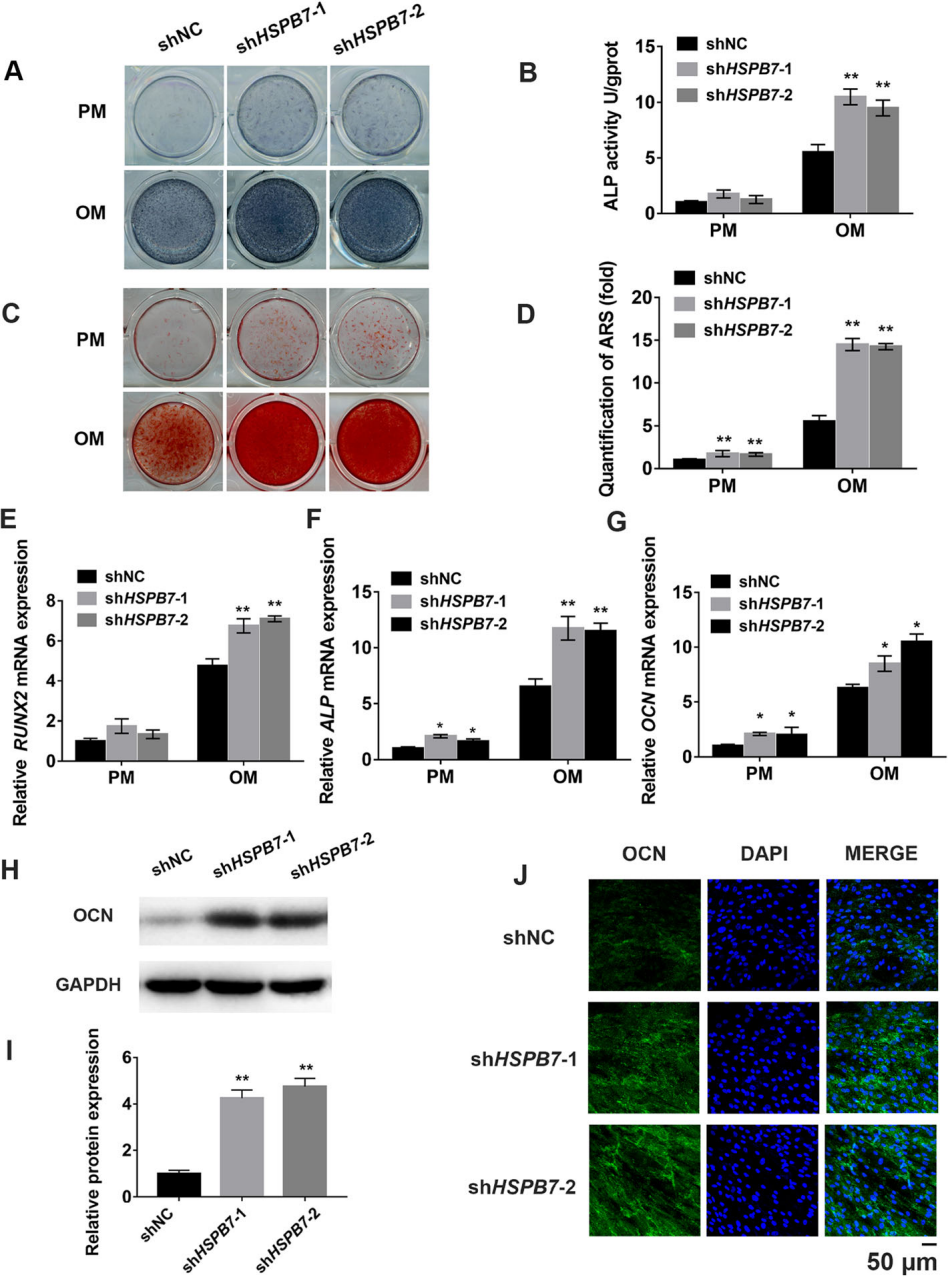
*HSPB7* knockdown promoted the osteogenic differentiation of hASCs in vitro. **a** ALP staining in the *HSPB7* knockdown (sh*HSPB7*-1, sh*HSPB7*-2) and control (shNC) groups at day 7 of osteogenic differentiation. **b** Histogram shows the 7-day quantification of ALP activity. **c** ARS staining at day 14 of osteogenic differentiation. **d** Relative quantitative analysis of ARS staining. **e**–**g** Relative mRNA expression of *RUNX2*, *ALP*, and *OCN* at day 14 of osteogenic differentiation. **h**, **i** *HSPB7* knockdown increased the protein expression of OCN. **j** Confocal microscopy confirmed the upregulation of OCN protein expression in *HSPB7* knockdown hASCs at day 14 of osteogenic differentiation. Scale bar = 50 μm. (*P < 0.05, **P < 0.01, compared with shNC)

The effect of *HSPB7* on osteogenesis was further evaluated by gene knockdown experiments using shRNA. To avoid off-target effect, two different shRNA sequences against *HSPB7* were employed. Following puromycin selection, the knockdown efficiency was verified by fluorescence staining (SFig. 2A), qRT-qPCR, and Western blot (SFig. 2E-G). As shown in Fig. 3a, b, ALP activity was significantly increased in the *HSPB7* knockdown group compared to the shNC group. Consistently, a greater number of mineralized nodules were observed in the *HSPB7* knockdown group than in the shNC group (Fig. 3c, d). Also, the mRNA expression levels of *RUNX2*, *ALP*, and *OCN* were increased after *HSPB7* knockdown (Fig. 3e–g). Additionally, *HSPB7* knockdown upregulated the protein expression of OCN (Fig. 3h–j).

To further validate the function of *HSPB7* in osteogenic differentiation, the recombinant human HSPB7 protein (rhHSPB7) was utilized for rescue experiments. Results showed the addition of rhHSPB7 successfully attenuated the osteogenic differentiation capacity of *HSPB7* knockdown cells as well as the shNC cells (SFig. 3A–G). In addition, we also evaluated the effect of *HSPB7* on osteogenic differentiation of hBMSCs. Similarly, *HSPB7* knockdown promoted osteogenesis of hBMSCs (SFig. 4A-F). Collectively, these findings suggested that *HSPB7* played a negative role in osteogenic differentiation.

### *HSPB7* knockdown activated ERK1/2 signaling pathway

**Fig. 4:**
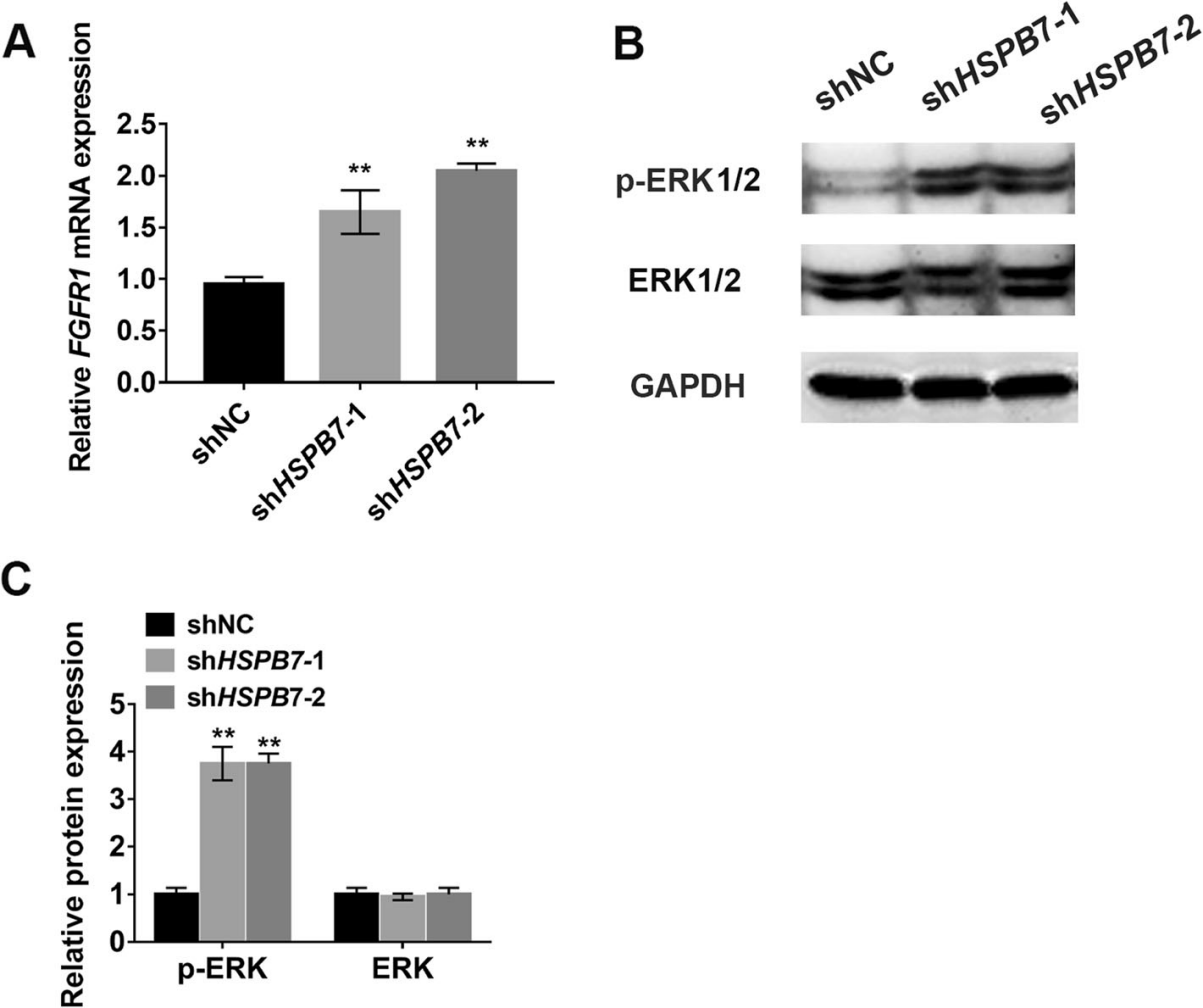
*HSPB7* knockdown activated ERK signaling pathway. **a** qRT-PCR showed *HSPB7* knockdown increased the expression of *FGFR1*. **b** *HSPB7* knockdown increased the level of phosphorylated ERK1/2 in hASCs. **c** The quantitative results of **b** by Image J software. Data are presented as the mean ± SD (**P < 0.01, compared with shNC)

To further explore the mechanism by which *HSPB7* regulates osteogenic differentiation, we examined several osteogenesis-related signaling pathways and found the expression of *FGFR1* and p-ERK1/2 was significantly upregulated after *HSPB7* knockdown (Fig. 4a–c). By contrast, the protein level of p-ERK1/2 was markedly decreased in *HSPB7* overexpressing hASCs (SFig. 5A and B). Due to the abovementioned results, we hypothesized that ERK signaling might be involved in *HSPB7*-mediated osteogenic differentiation.

### *HSPB7* knockdown promoted osteogenesis through ERK1/2 signaling pathway

**Fig. 5:**
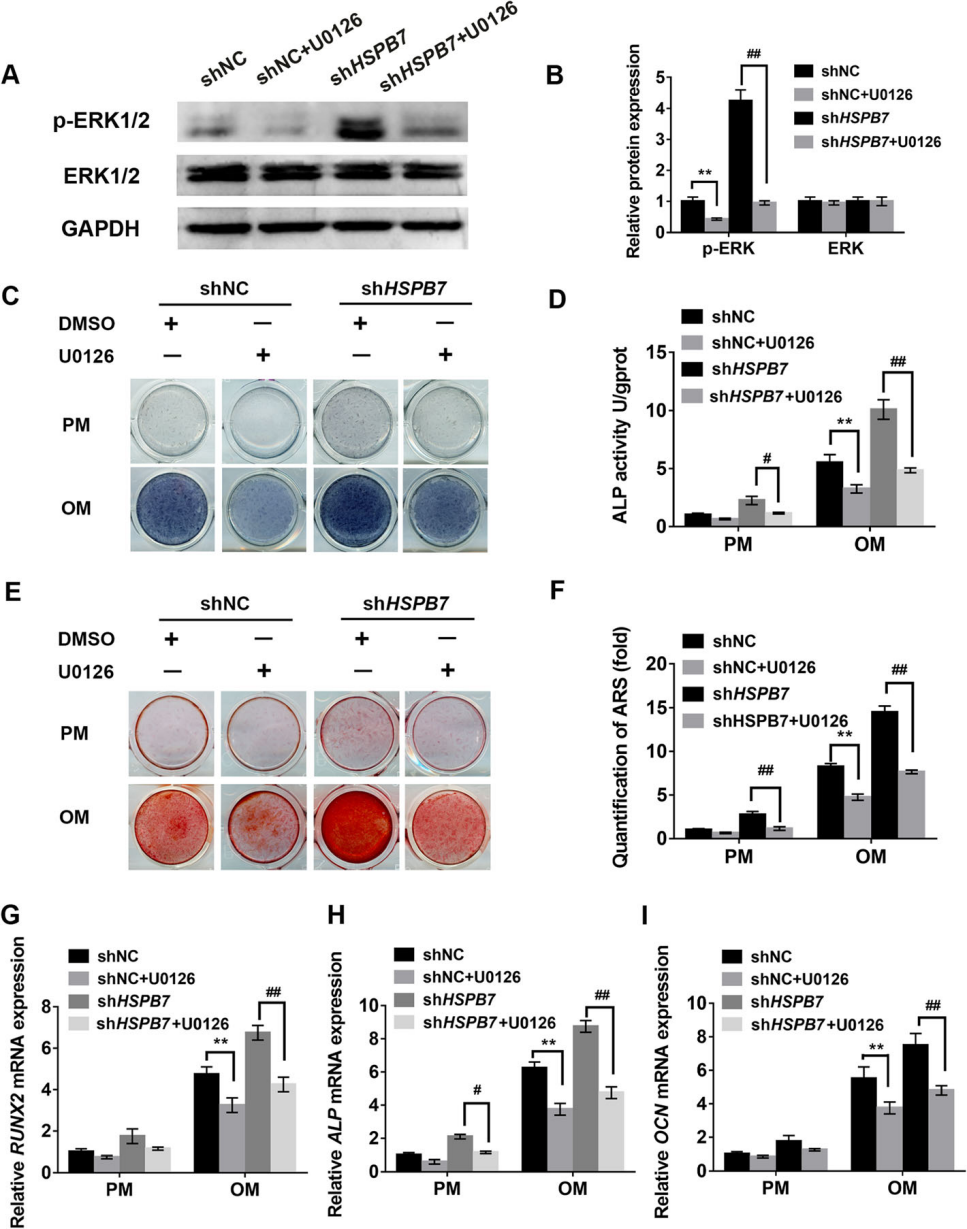
Inhibiting ERK signaling pathway with U0126 reversed the enhancing effect of *HSPB7* knockdown on osteogenesis of hASCs. **a** U0126 reduced the level of phosphorylated ERK1/2 in shNC and sh*HSPB7* hASCs. **b** The quantitative results of **a** by Image J software. **c** ALP staining at day 7 of osteogenic differentiation. U0126 (10 μM) or DMSO was incubated for 7 days. **d** Histogram shows the 7-day quantification of ALP activity. **e** ARS staining in shNC, sh*HSPB7* groups treated with U0126 (10 μM) or DMSO (control) for 14 days. **f** Relative quantitative analysis of ARS staining. **g**–**i** Relative mRNA expression of *RUNX2*, *ALP*, and OCN at day 14 of osteogenic differentiation in the presence or absence of U0126 (10 μM). DMSO was used as a control. Data are presented as the mean ± SD (*P < 0.05, **P < 0.01, compared with shNC)

To verify whether *HSPB7* regulated osteogenesis via ERK1/2 signaling pathway, we examined the inhibitory effect of ERK1/2 signaling on osteogenic differentiation in the *HSPB7* knockdown group. Western blot showed the level of p-ERK1/2 was significantly inhibited with the addition of U0126 (Fig. 5a, b). Following treatment with U0126, the increased ALP activity induced by *HSPB7* knockdown was effectively abrogated (Fig. 5c, d). Meanwhile, the extracellular matrix mineralization was also reduced in *HSPB7* knockdown hASCs in the presence of U0126 (Fig. 5e, f). Moreover, the upregulated mRNA expression levels of osteogenic markers *RUNX2*, *ALP*, and *OCN* caused by *HSPB7* silencing were diminished with the addition of U0126 (Fig. 5g–i).

Next, ERK1/2 siRNA was used to confirm whether ERK1/2 knockdown could block the increased osteogenic ability induced by *HSPB7* deficiency. The knockdown efficiency of ERK1/2 was demonstrated by Western blot (SFig. 6A and B). As expected, the promotive effect of *HSPB7* knockdown on osteogenesis was markedly reversed in the *HSPB7* and ERK1/2 double knockdown hASCs, which was revealed by ALP staining and quantification (SFig. 6C and D), ARS staining and quantification (SFig. 6E and F), and qRT-PCR (SFig. 6G-I). Taken together, these results suggested that *HSPB7* regulated osteogenic differentiation through ERK signaling pathway.

Although we found *HSPB7* regulated osteogenesis with an involvement of ERK signaling pathway, we cannot exclude the possibility that other signaling pathways are also involved in *HSPB7*-mediated osteogenic differentiation. In this study, we also found a negative correlation between the mRNA expression of *HSPB7* and *BMPRI/II* (SFig. 7). These data suggested that *BMPRI/II* might be implicated in *HSPB7*-regulated osteogenesis. Future investigation is needed to elucidate the mechanism.

### *HSPB7* inhibited bone formation of hASCs in vivo

**Fig. 6:**
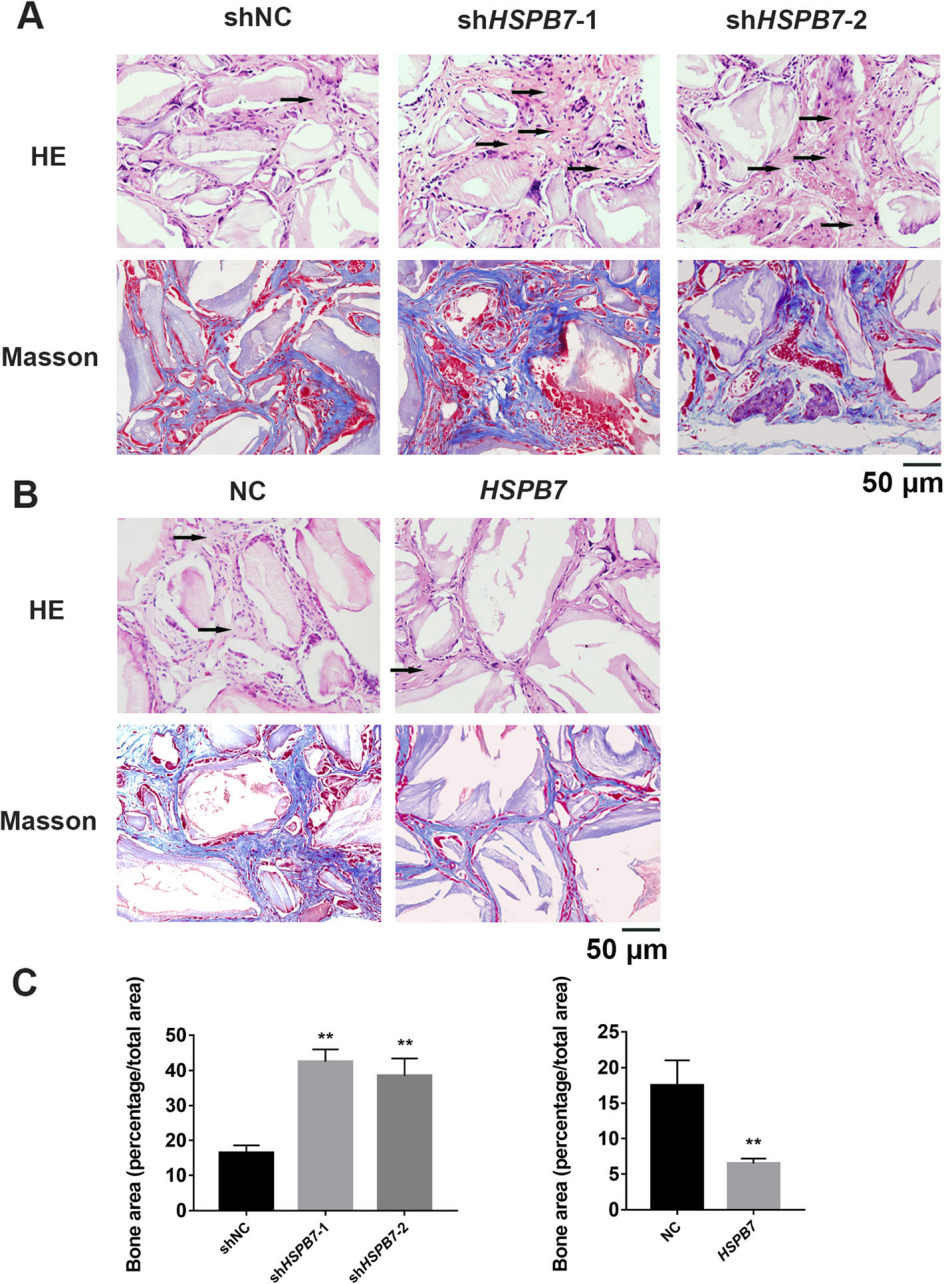
*HSPB7* knockdown promoted bone formation of hASCs in vivo. **a** *HSPB7* knockdown promoted bone formation capacity of hASCs in vivo, as determined by HE staining (HE) and Masson’s trichrome staining (Masson). **b** *HSPB7* overexpression inhibited bone formation capacity of hASCs. **c** Quantitative measurements of bone-like tissue showed the area of bone-like tissue was significantly increased in *HSPB7* knockdown hASCs but decreased in *HSPB7* overexpression hASCs. Black arrows indicate new bone-like tissue. Scale bar = 50 μm. Data are presented as the mean ± SD (**P < 0.01, compared with shNC or NC)

To validate our findings in vitro, we examined whether *HSPB7* could affect the bone formation ability of hASCs in vivo. As shown in Fig. 6a, HE staining and Masson’s trichrome staining revealed the amount of newly generated bone was higher in *HSPB7* knockdown hASCs compared with the control cells. On the contrary, mice implanted with *HSPB7* overexpression hASCs showed much less bone-like tissue (Fig. 6b). Furthermore, quantitative measurements using SPOT 4.0 software confirmed that the volume of newly formed bone was increased in *HSPB7* knockdown hASCs but reduced in *HSPB7* overexpression hASCs (Fig. 6c). These results indicated that *HSPB7* might suppress osteogenic differentiation of hASCs in vivo.

### *HSPB7* promoted adipogenesis of hASCs

It has been reported that osteogenesis and adipogenesis of MSCs are competing and reciprocal [22]. Thus, we also investigated the role of *HSPB7* in adipogenesis. We observed that *HSPB7* was upregulated during adipogenic differentiation of hASCs (SFig. 8A). Oil Red O staining and qRT-PCR showed that *HSPB7* overexpression significantly promoted adipogenic differentiation of hASCs (SFig. 8B-D).

### *HSPB7* was increased in OVX mice

The imbalance between osteogenesis and adipogenesis is associated with various bone-related diseases, such as osteoporosis. Based on above results, it was reasonable to hypothesize that *HSPB7* might be abnormally expressed in osteoporotic mice. To verify this hypothesis, we constructed osteoporotic mouse models by ovariectomy. HE staining and micro-CT showed that the trabecular number (Tb.N) and bone volume/total volume (BV/TV) were significantly reduced, whereas the trabecular separation (Tb.SP) was increased in OVX mice compared to those in SHAM mice, which confirmed the establishment of osteoporotic models (SFig. 9A-D). Then, mBMSCs were collected from OVX and SHAM mice and identified by flow cytometry. The protein expression of HSPB7 was greatly increased in mBMSCs from osteoporotic mice, as revealed by Western blot (SFig. 9E and F). These data suggested that *HSPB7* might be involved in the pathogenesis of osteoporosis. More experimental studies are needed to further explore the role of *HSPB7* in osteoporosis.

## Discussion

During recent years, hASCs isolated from adipose tissue have emerged as an attractive cell source for bone tissue engineering which sheds a light on bone repair and regeneration [23–25]. However, employing hASCs to promote bone repair is far from being applied in clinical routine due to the limited knowledge about osteogenic differentiation. Thus, it is of great significance to investigate the molecular basis of osteogenesis. Herein, we demonstrated that *HSPB7* was markedly reduced during osteogenesis of hASCs and *HSPB7* knockdown enhanced osteogenic capacity of hASCs both in vitro and in vivo.

*HSPB7*, located on 1p36.13, is a member of heat shock protein (HSP) family that plays essential roles in protein folding and cellular protein homeostasis [26]. Heat shock proteins have been implicated in diverse range of cellular processes, such as stress response, cell proliferation, and apoptosis [27–29]. Recent studies have revealed that heat shock proteins also play crucial roles in osteogenic differentiation. Flanagan et al. reported that heat shock protein B8 (*HSPB8*) knockdown significantly reduced osteogenic differentiation potential of dental pulp stem cells [30]. Chen et al. showed extracellular heat shock protein 70 (*HSP70*) enhanced osteogenesis of human mesenchymal stem cells (hMSCs) [31]. A study by Zhang et al. demonstrated low-intensity pulsed ultrasound stimulated osteogenesis of hASCs possibly by increasing the expression of *HSP70* and *HSP90* [32]. *HSPB7*, which is highly expressed in the heart, has been intensively investigated in multiple cardiac pathologies [33]. In the present study, our results provided novel insight into how *HSPB7* regulated osteogenic differentiation of hASCs. We first showed that *HSPB7* knockdown enhanced hASC osteogenesis through ERK signaling.

Extracellular signal-regulated kinase (ERK) pathway, also known as the Ras-Raf-MEK-ERK pathway, is involved in a variety of physiological processes such as cell proliferation, division, and survival [34, 35]. Numerous studies have revealed that ERK signaling pathway plays vital roles in the osteogenic differentiation [36–39]. With regard to the relationship between *HSPB7* and ERK signaling pathway, Naderi found *HSPB7* overexpression in breast cancer cells reduced the level of p-ERK [18]. In agreement with the above study, we found *HSPB7* overexpression reduced the level of p-ERK, while *HSPB7* knockdown significantly activated ERK signaling pathway. To clarify the involvement of ERK signaling pathway in *HSPB7*-mediated osteogenic differentiation of hASCs, we examined whether inhibiting ERK signaling pathway with U0126 or siERK1/2 could attenuate the effect of *HSPB7* knockdown on osteogenesis of hASCs. Results showed inhibition of ERK signaling pathway effectively blocked the enhancing effect of *HSPB7* knockdown on osteogenesis. Although our study demonstrated that ERK signaling pathway played a crucial role in osteogenesis induced by *HSPB7* knockdown, it is likely that other signaling pathways are also involved in this process. Previous studies indicated that *HSPB7* was associated with diverse important signaling pathways. For example, Naderi found *HSPB7* overexpression could downregulate the expression of p-AKT which is closely associated with osteogenic differentiation [18]. In this study, we observed the expression of *BMPRI/II* was negatively regulated by *HSPB7*, which suggested that *BMPRI/II* might be involved in *HSPB7*-regulated osteogenesis. Future studies are still needed to elucidate the mechanism of *HSPB7*-mediated osteogenesis.

Osteoporosis is a major public health problem which has a high incidence and great risk of fracture. To date, molecular pathways involved in the pathogenesis of osteoporosis remain elusive. Uncovering the mechanisms underlying osteoporosis is important for developing novel therapies and effective prevention strategies [40]. It has been well established that the imbalance between adipogenesis and osteogenesis contributes to osteoporosis [41]. In this study, we found *HSPB7* expression was significantly increased during adipogenic differentiation of hASCs. We hypothesized that *HSPB7* might be aberrantly expressed in osteoporosis model. Therefore, we constructed OVX mice and examined *HSPB7* expression in mBMSCs from OVX. As expected, we found the protein expression of HSPB7 was significantly increased in OVX mice, which suggested the potential involvement of *HSPB7* in the pathogenesis of osteoporosis. The present study provides new insight into the molecular basis of osteoporosis and implied that *HSPB7* might be used as a potential diagnostic marker and therapeutic target for osteoporosis.

## Conclusions

**Fig. 7:**
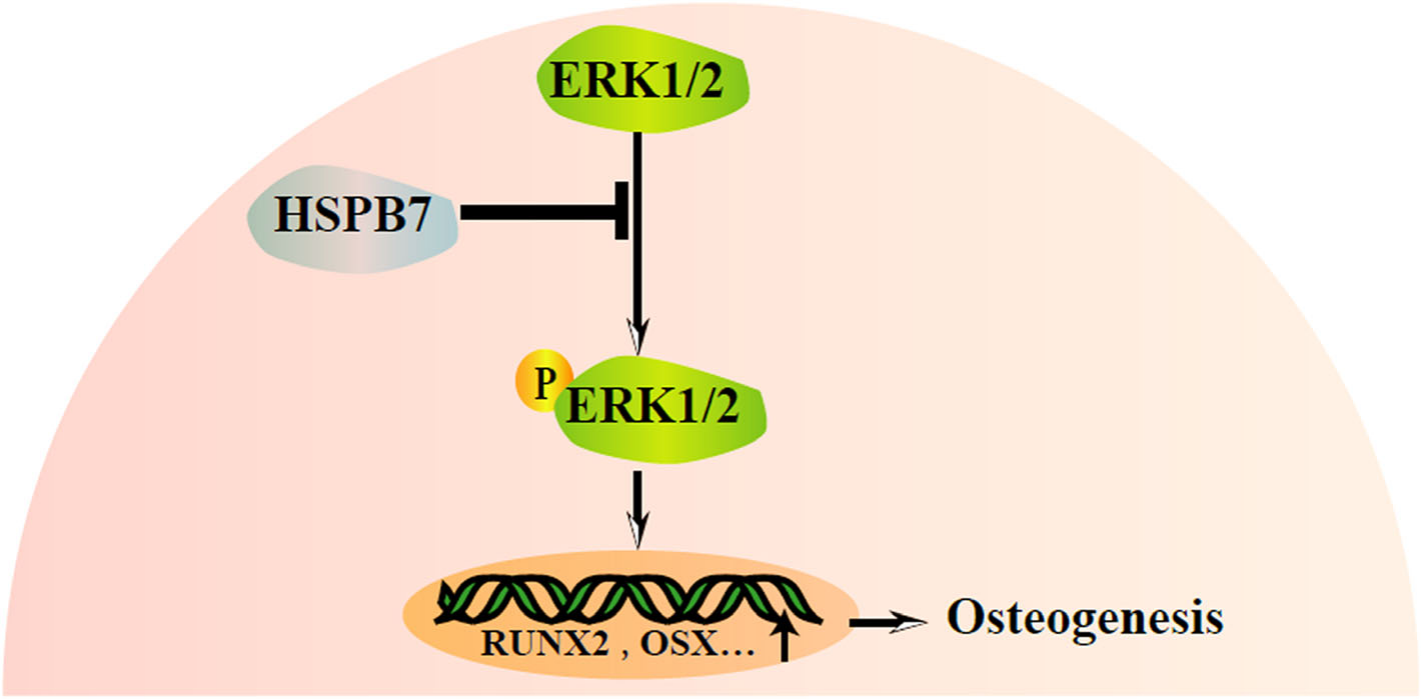
Schematic diagram of the regulation of osteogenesis by *HSPB7*. *HSPB7* regulated the osteogenic differentiation of hASCs via ERK1/2 signaling

In summary, this is the first study to identify the negative role of *HSPB7* in osteogenic differentiation of hASCs with an involvement of ERK signaling pathway (Fig. 7). These findings could provide not only new ideas for exploring new approaches in stimulating osteogenesis of hASCs but also a potential therapeutic target for the skeletal diseases.

## Supplementary information

**Additional file 1: Supplementary Figure 1.** The expression of *HSPB7* in different cell types. (A) The mRNA expression of *HSPB7* in hASCs, hBMSCs and U2OS cells. (B) The mRNA expression of *Hspb7* in mBMSCs and MC3T3-E1 cells. (C) The mRNA expression level of *HSPB7* was upregulated with passage in hASCs. Results are presented as the mean ± SD. (*P < 0.05, **P < 0.01).

**Additional file 2: Supplementary Figure 2.** Lentiviral transfection to overexpress or knockdown *HSPB7* in hASCs. (A) Images of GFP-positive hASCs under a normal microscope and a fluorescence microscope. Scale bar = 200 μm. (B) Relative mRNA expression of *HSPB7* in NC, *HSPB7* groups. (C, D) Protein levels of HSPB7 in the *HSPB7* overexpression group (*HSPB7*) and negative control (NC) group. (E) Relative mRNA expression of *HSPB7* in shNC, sh*HSPB7*-1 and sh*HSPB7*-2 groups. (F, G) Protein levels of HSPB7 in the *HSPB7* knockdown group (sh*HSPB7*-1, sh*HSPB7*-2) and negative control (shNC) group. GAPDH was used as an internal control. Results are presented as the mean ± SD. (**P < 0.01, compared with NC or shNC).

**Additional file 3: Supplementary Figure 3.** Recombinant human HSPB7 protein reversed the enhancing effect of *HSPB7* knockdown on osteogenesis of hASCs. (A, B) ALP activity of shNC and sh*HSPB7* hASCs at day 7 in the presence or absence of recombinant human HSPB7 (rhHSPB7, 400 ng/ml). Equal volume of PBS was used as a control. (C.D) ARS staining and quantification at day 14. (E-G) Relative mRNA levels of *RUNX*2, *ALP* and *OCN* determined by qRT-PCR at day 14 after osteogenic induction. Results are presented as the mean ± SD. (*/^#^ *P*<0.05, **/^##^*P* < 0.01, *compared with shNC, ^#^compared with sh*HSPB7*).

**Additional file 4: Supplementary Figure 4.** *HSPB7* knockdown promoted osteogenesis of hBMSCs. (A) qRT-PCR showed that the mRNA expression of *HSPB7* was decreased during the osteogenic differentiation of hBMSCs. (B) The knockdown efficiency of *HSPB7* in hBMSCs. (C) ALP staining in *HSPB7* knockdown (sh*HSPB7-*1, sh*HSPB7-*2) and control (shNC) groups on day 7 after osteogenic induction. (D) Relative quantitative analyses of ALP activity on day 7 after osteogenic induction. (E) ARS staining on day 14 after osteogenic induction. (F) Relative quantitative analysis of ARS staining. Results are presented as the mean ± SD. (*P < 0.05, **P < 0.01, compared with day 0 or shNC).

**Additional file 5: Supplementary Figure 5.** *HSPB7* overexpression inhibited ERK signalling pathway. (A) *HSPB7* overexpression reduced the level of phosphorylated ERK1/2 in hASCs. (B) The quantitative results of (A) by Image J software. Data are presented as the mean ± SD (**P < 0.01, compared with NC).

**Additional file 6: Supplementary Figure 6.** Knockdown of ERK1/2 blocked the promotive effect of *HSPB7* knockdown on osteogenesis of hASCs. (A, B) The knockdown efficiency of ERK1/2 was verified by Western blot. (C, D) ALP activity was significantly reduced in *HSPB7/ERK1/2* double knockdown hASCs in comparison with *HSPB7* knockdown hASCs. (E, F) ARS staining and quantification at day 14 of osteogenic differentiation. (G-I) Relative mRNA expression of *RUNX2*, *ALP* and *OCN* at day 14 of osteogenic differentiation. Results are presented as the mean ± SD. (*/^#^*P* < 0.05, **/^##^*P* < 0.01, *compared with shNC+siNC, ^#^ compared with sh*HSPB7*+siNC).

**Additional file 7: Supplementary Figure 7.** *HSPB7* regulated *BMPRI/II e*xpression. qRT-PCR analysis showed that *HSPB7* knockdown significantly upregulated the mRNA expression of *BMPRIA* (A), *BMPRIB* (B) and *BMPRII* (C), whereas *HSPB7* overexpression downregulated the expression of *BMPRIA, BMPRIB* and *BMPRII*. Results are presented as the mean ± SD. (*P < 0.05, **P < 0.01, compared with shNC).

**Additional file 8: Supplementary Figure 8.** *HSPB7* regulated adipogenesis of hASCs. (A) The mRNA expression of *HSPB7* was increased during the adipogenic differentiation of hASCs, as detected by qRT-PCR. (B) Lentivirus transfection was conducted to overexpress *HSPB7* in hASCs and confirmed by qRT-PCR. (C) Cells were treated with proliferation medium (PM) or adipogenic medium (AM) for 14 days. *HSPB7* overexpression increased the lipid accumulation of hASCs, as revealed by Oil red O staining. (D) *HSPB7* overexpression promoted the expression of adipogenic marker gene *PPARγ*, as determined by qRT-PCR. Results are presented as the mean ± SD. (*P < 0.05, **P < 0.01, compared with NC).

**Additional file 9: Supplementary Figure 9.** *HSPB7* expression was increased in mBMSCs from OVX mice. (A) Representative HE staining and Micro CT images at 12 weeks after ovariectomy. Scale bars for HE staining and Micro CT represent 50 μm and 1mm, respectively. (B-D) Quantitative analyses of parameters regarding bone microstructure, including trabecular number (Tb.N), trabecular bone volume/tissue volume (BV/TV), and trabecular spacing (Tb.Sp). (E) Western blot analysis showed that HSPB7 expression was significantly increased in mBMSCs from OVX mice compared with SHAM mice. (F) The band intensities of (E) were analyzed by Image J software. GAPDH was used as the internal control. Results are presented as the mean ± SD. (**P < 0.01, compared with SHAM group).

## Data Availability

The authors confirm that all data underlying the findings are fully available.

## Abbreviations

hASCs: Human adipose derived stem cells
hBMSCs: Human bone marrow mesenchymal stem cells
mBMSCs: Mouse bone marrow mesenchymal stem cells
PM: Proliferation medium
OM: Osteogenic medium
AM: Adipogenic medium
siRNA: Small interfering RNA
NC: Negative control
HE: Hematoxylin and eosin
ALP: Alkaline phosphatase
ARS: Alizarin Red S
qRT-PCR: Quantitative reverse transcription polymerase chain reaction
GAPDH: Glyceraldehyde 3-phosphatedehydrogenase
OCN: Osteocalcin
RUNX2: Runt-related transcription factor 2
PBS: Phosphate-buffered saline
TBST: Tris-buffered saline Tween-20
p-ERK1/2: Phosphorylated extracellular signal-regulated kinase1/2
rhHSPB7: Recombinant human HSPB7 protein
