# Supplementary figures and images for: HSPB7 regulates osteogenic differentiation of human adipose derived stem cells via ERK signaling pathway
Source: Stem Cell Res Ther. 2020 Oct 23;11:450. doi: 10.1186/s13287-020-01965-4 (PMC7583167; doi:10.1186/s13287-020-01965-4)

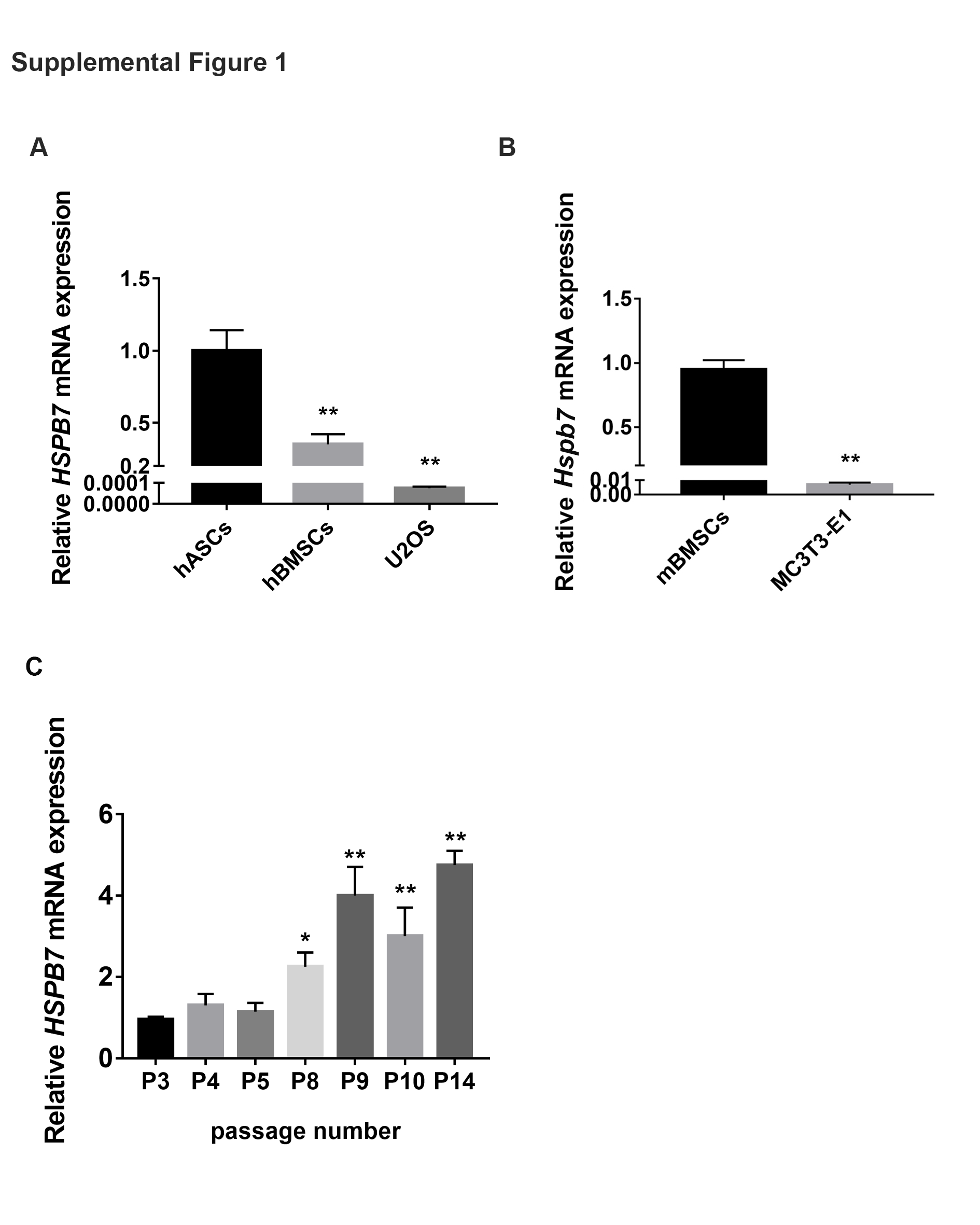

Supplement: Supplementary file 1 — Additional file 1: Supplementary Figure 1. The expression of HSPB7 in different cell types. (A) The mRNA expression of HSPB7 in hASCs, hBMSCs and U2OS cells. (B) The mRNA expression of Hspb7 in mBMSCs and MC3T3-E1 cells. (C) The mRNA expression level of HSPB7 was upregulated with passage in hASCs. Results are presented as the mean ± SD. (*P < 0.05, **P < 0.01). [file 13287_2020_1965_MOESM1_ESM.tif]

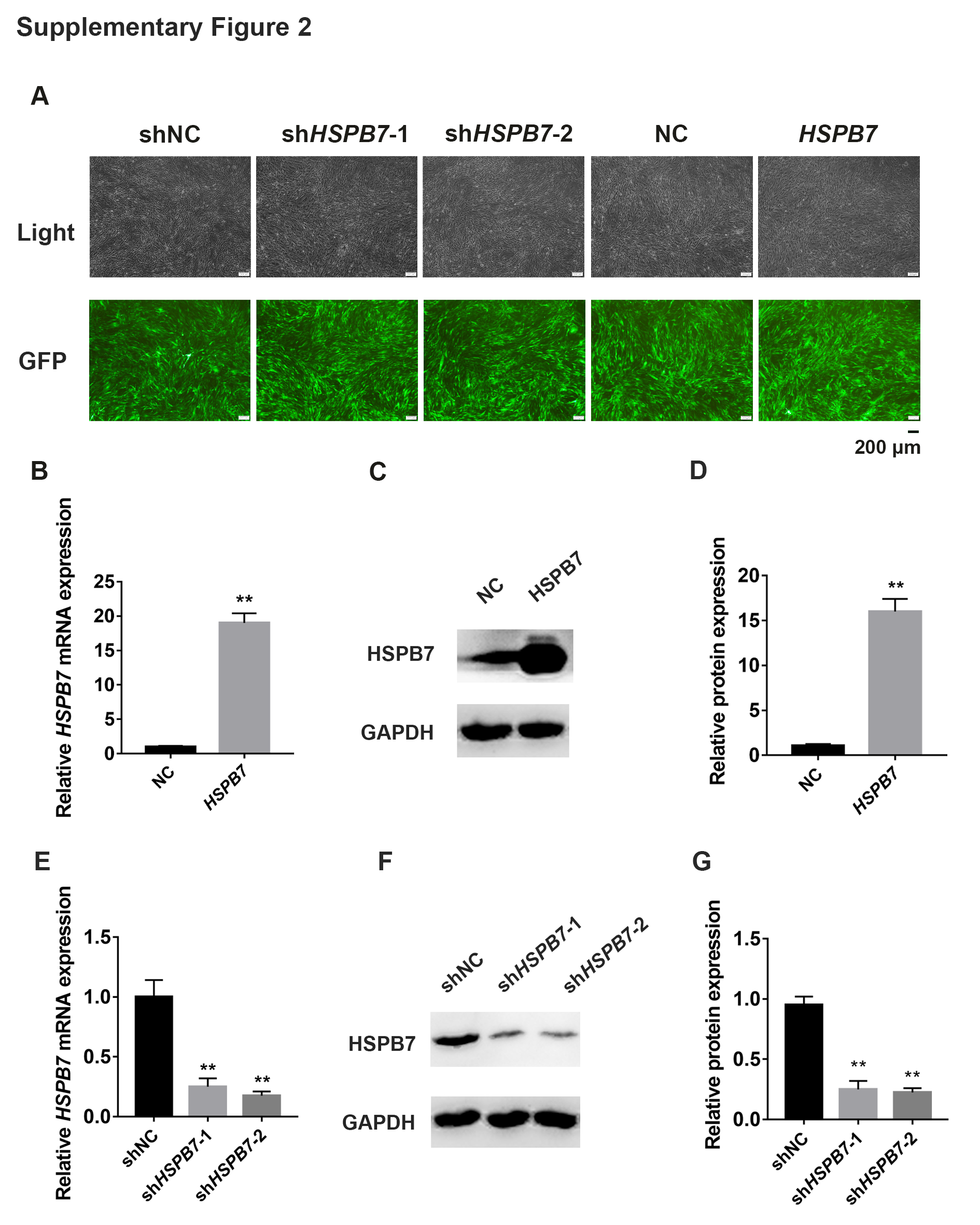

Supplement: Supplementary file 2 — Additional file 2: Supplementary Figure 2. Lentiviral transfection to overexpress or knockdown HSPB7 in hASCs. (A) Images of GFP-positive hASCs under a normal microscope and a fluorescence microscope. Scale bar = 200 μm. (B) Relative mRNA expression of HSPB7 in NC, HSPB7 groups. (C, D) Protein levels of HSPB7 in the HSPB7 overexpression group (HSPB7) and negative control (NC) group. (E) Relative mRNA expression of HSPB7 in shNC, shHSPB7-1 and shHSPB7-2 groups. (F, G) Protein levels of HSPB7 in the HSPB7 knockdown group (shHSPB7-1, shHSPB7-2) and negative control (shNC) group. GAPDH was used as an internal control. Results are presented as the mean ± SD. (**P < 0.01, compared with NC or shNC). [file 13287_2020_1965_MOESM2_ESM.tif]

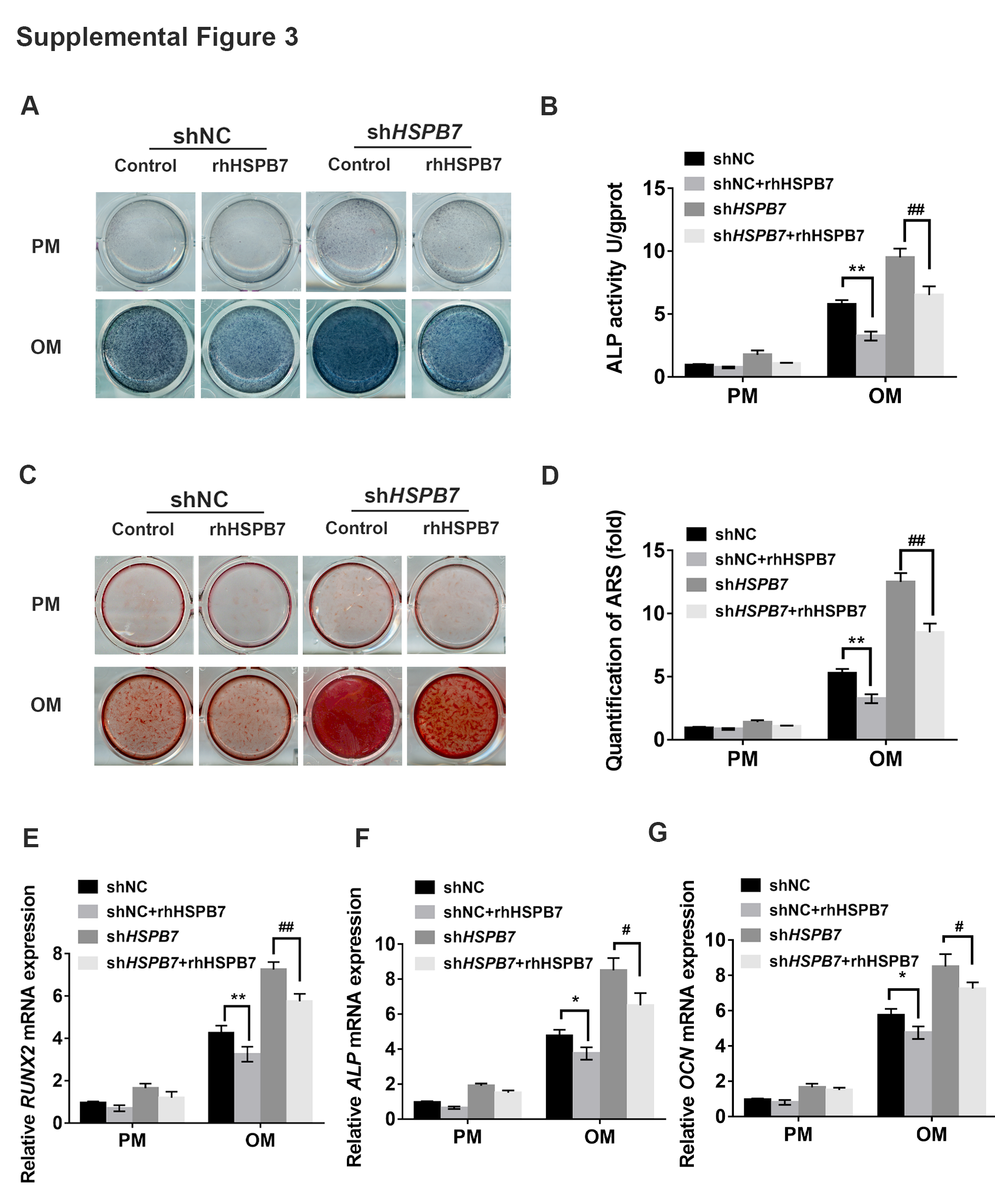

Supplement: Supplementary file 3 — Additional file 3: Supplementary Figure 3. Recombinant human HSPB7 protein reversed the enhancing effect of HSPB7 knockdown on osteogenesis of hASCs. (A, B) ALP activity of shNC and shHSPB7 hASCs at day 7 in the presence or absence of recombinant human HSPB7 (rhHSPB7, 400 ng/ml). Equal volume of PBS was used as a control. (C.D) ARS staining and quantification at day 14. (E-G) Relative mRNA levels of RUNX2, ALP and OCN determined by qRT-PCR at day 14 after osteogenic induction. Results are presented as the mean ± SD. (*/# P<0.05, **/##P < 0.01, *compared with shNC, #compared with shHSPB7). [file 13287_2020_1965_MOESM3_ESM.tif]

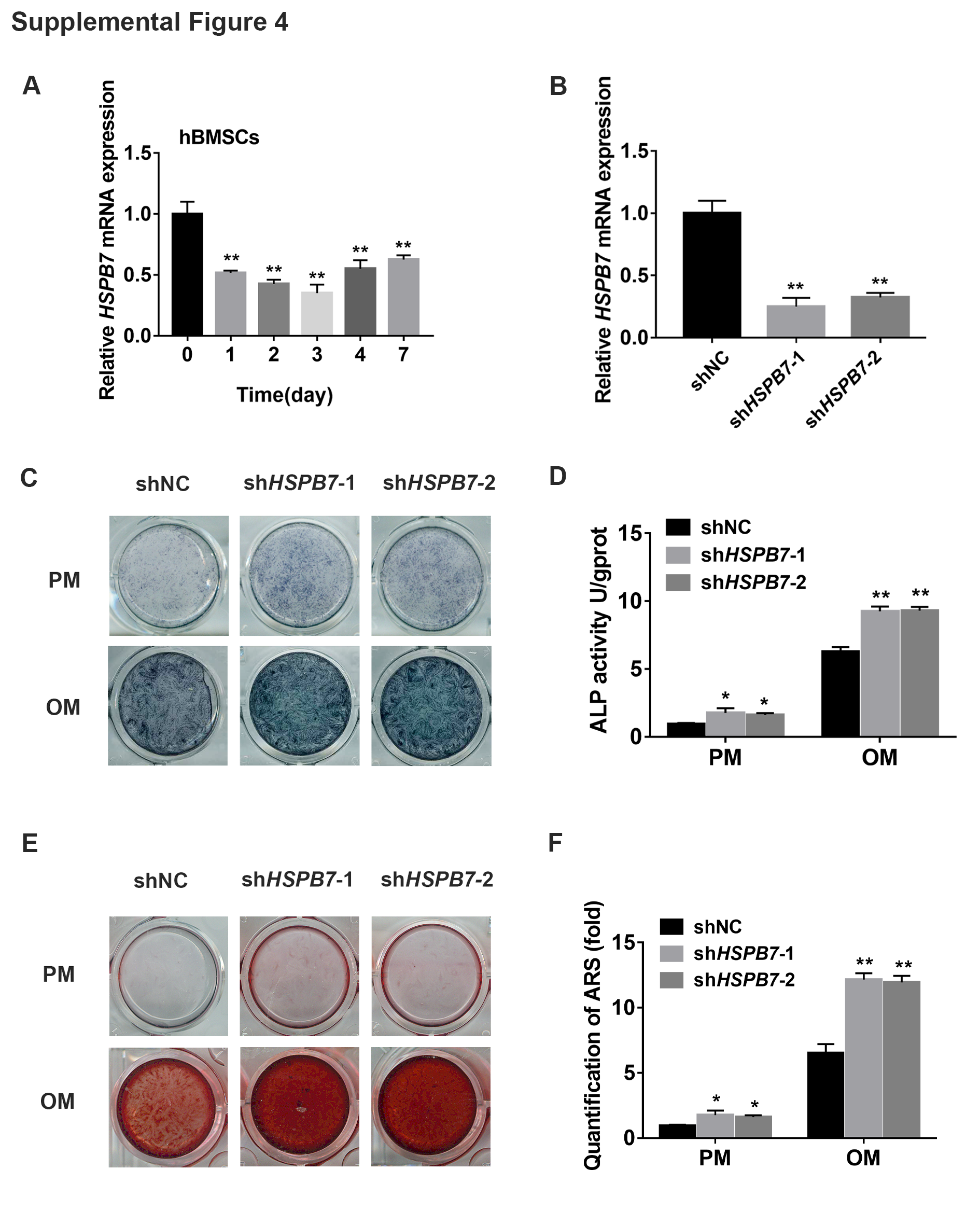

Supplement: Supplementary file 4 — Additional file 4: Supplementary Figure 4. HSPB7 knockdown promoted osteogenesis of hBMSCs. (A) qRT-PCR showed that the mRNA expression of HSPB7 was decreased during the osteogenic differentiation of hBMSCs. (B) The knockdown efficiency of HSPB7 in hBMSCs. (C) ALP staining in HSPB7 knockdown (shHSPB7-1, shHSPB7-2) and control (shNC) groups on day 7 after osteogenic induction. (D) Relative quantitative analyses of ALP activity on day 7 after osteogenic induction. (E) ARS staining on day 14 after osteogenic induction. (F) Relative quantitative analysis of ARS staining. Results are presented as the mean ± SD. (*P < 0.05, **P < 0.01, compared with day 0 or shNC). [file 13287_2020_1965_MOESM4_ESM.tif]

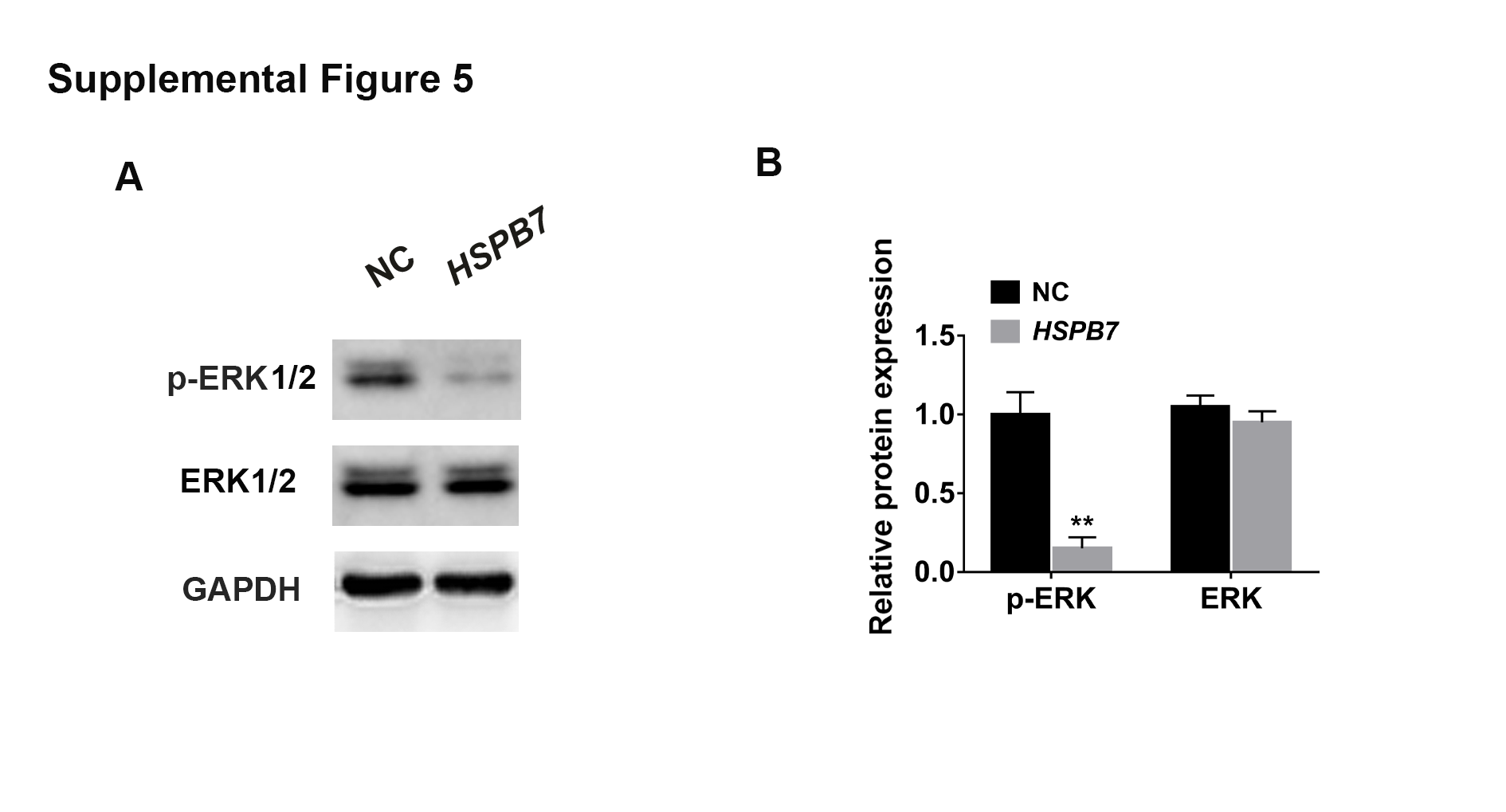

Supplement: Supplementary file 5 — Additional file 5: Supplementary Figure 5. HSPB7 overexpression inhibited ERK signalling pathway. (A) HSPB7 overexpression reduced the level of phosphorylated ERK1/2 in hASCs. (B) The quantitative results of (A) by Image J software. Data are presented as the mean ± SD (**P < 0.01, compared with NC). [file 13287_2020_1965_MOESM5_ESM.tif]

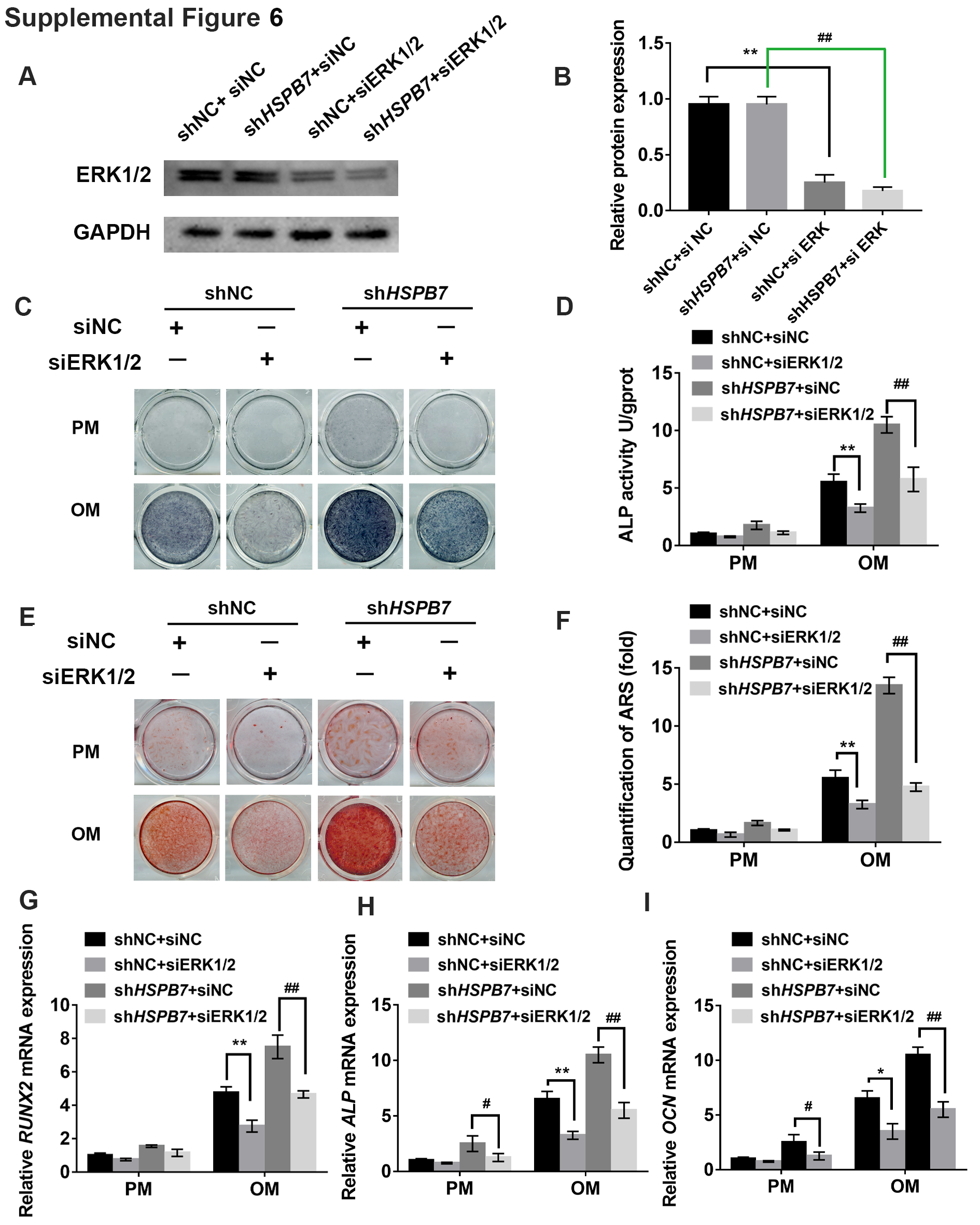

Supplement: Supplementary file 6 — Additional file 6: Supplementary Figure 6. Knockdown of ERK1/2 blocked the promotive effect of HSPB7 knockdown on osteogenesis of hASCs. (A, B) The knockdown efficiency of ERK1/2 was verified by Western blot. (C, D) ALP activity was significantly reduced in HSPB7/ERK1/2 double knockdown hASCs in comparison with HSPB7 knockdown hASCs. (E, F) ARS staining and quantification at day 14 of osteogenic differentiation. (G-I) Relative mRNA expression of RUNX2, ALP and OCN at day 14 of osteogenic differentiation. Results are presented as the mean ± SD. (*/#P < 0.05, **/##P < 0.01, *compared with shNC+siNC, # compared with shHSPB7+siNC). [file 13287_2020_1965_MOESM6_ESM.tif]

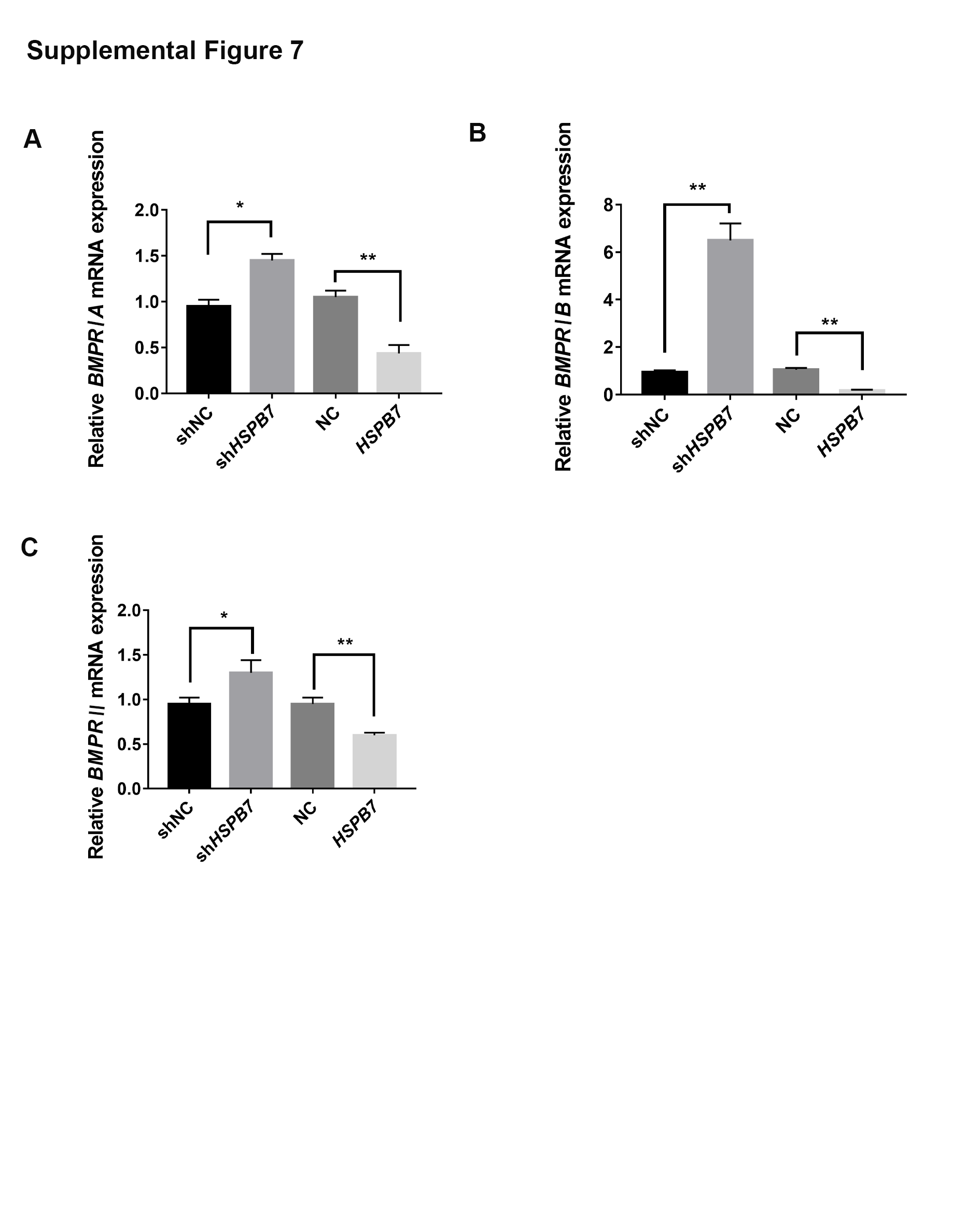

Supplement: Supplementary file 7 — Additional file 7: Supplementary Figure 7. HSPB7 regulated BMPRI/II expression. qRT-PCR analysis showed that HSPB7 knockdown significantly upregulated the mRNA expression of BMPRIA (A), BMPRIB (B) and BMPRII (C), whereas HSPB7 overexpression downregulated the expression of BMPRIA, BMPRIB and BMPRII. Results are presented as the mean ± SD. (*P < 0.05, **P < 0.01, compared with shNC). [file 13287_2020_1965_MOESM7_ESM.tif]

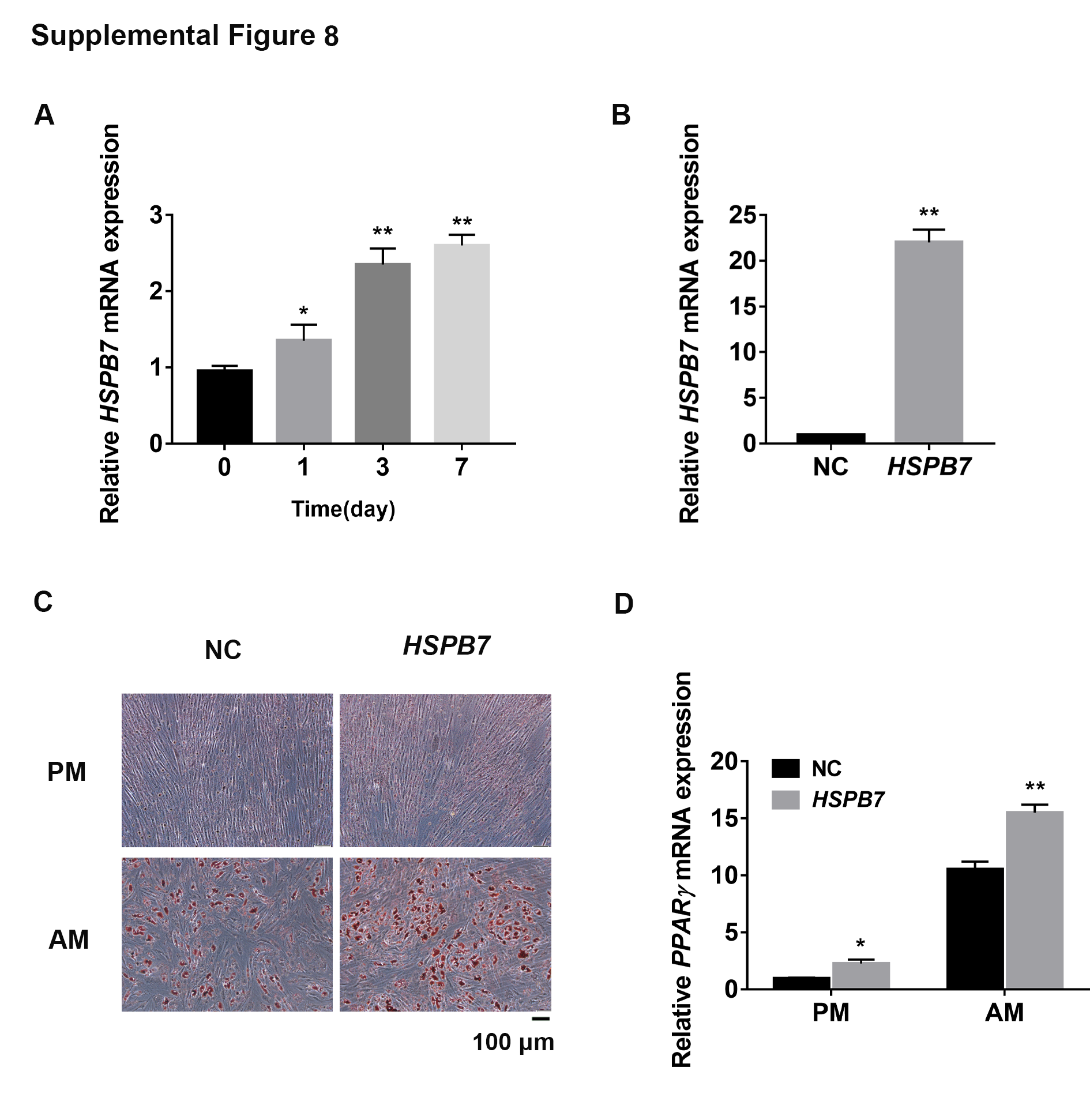

Supplement: Supplementary file 8 — Additional file 8: Supplementary Figure 8. HSPB7 regulated adipogenesis of hASCs. (A) The mRNA expression of HSPB7 was increased during the adipogenic differentiation of hASCs, as detected by qRT-PCR. (B) Lentivirus transfection was conducted to overexpress HSPB7 in hASCs and confirmed by qRT-PCR. (C) Cells were treated with proliferation medium (PM) or adipogenic medium (AM) for 14 days. HSPB7 overexpression increased the lipid accumulation of hASCs, as revealed by Oil red O staining. (D) HSPB7 overexpression promoted the expression of adipogenic marker gene PPARγ, as determined by qRT-PCR. Results are presented as the mean ± SD. (*P < 0.05, **P < 0.01, compared with NC). [file 13287_2020_1965_MOESM8_ESM.tif]

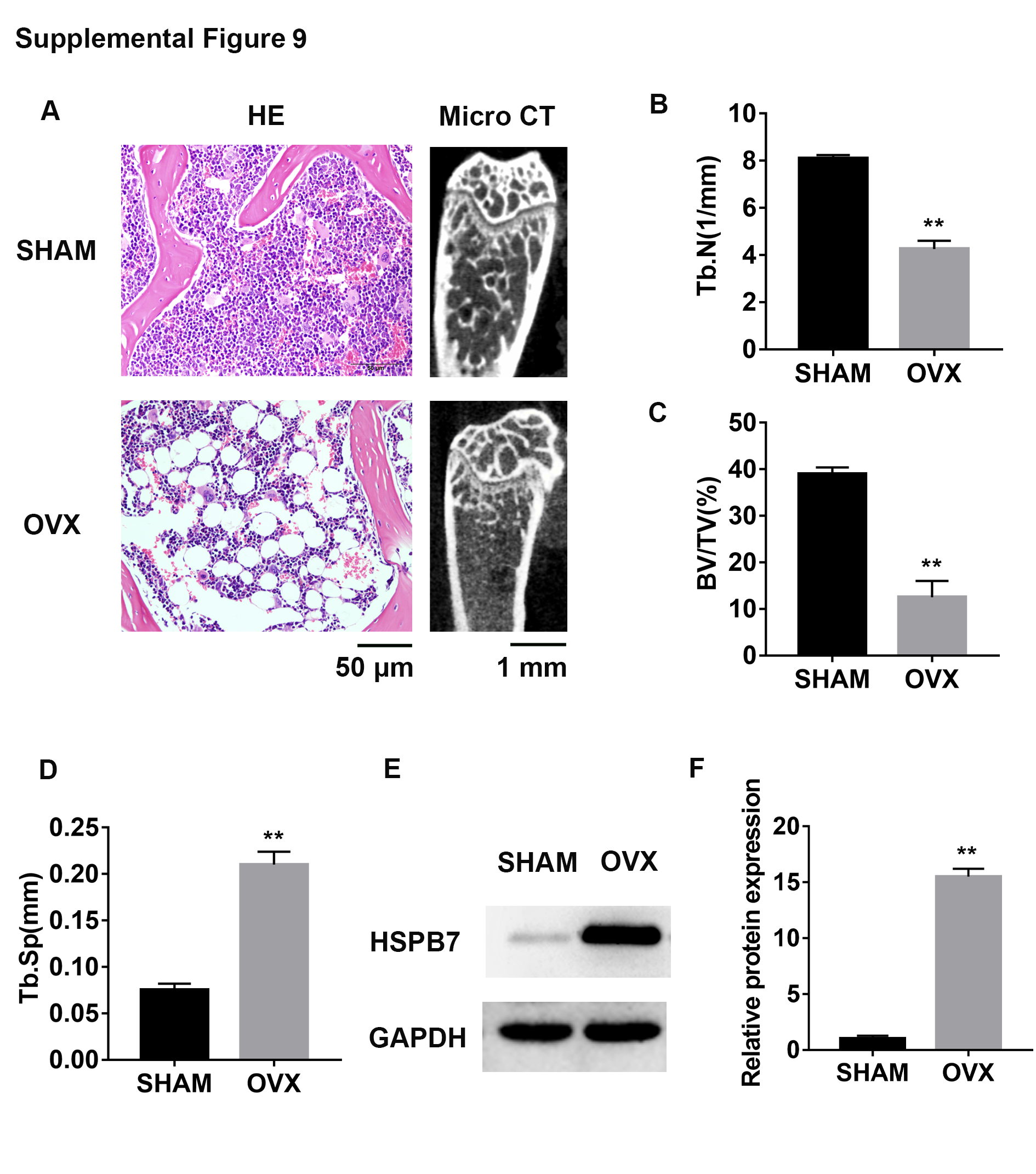

Supplement: Supplementary file 9 — Additional file 9: Supplementary Figure 9. HSPB7 expression was increased in mBMSCs from OVX mice. (A) Representative HE staining and Micro CT images at 12 weeks after ovariectomy. Scale bars for HE staining and Micro CT represent 50 μm and 1mm, respectively. (B-D) Quantitative analyses of parameters regarding bone microstructure, including trabecular number (Tb.N), trabecular bone volume/tissue volume (BV/TV), and trabecular spacing (Tb.Sp). (E) Western blot analysis showed that HSPB7 expression was significantly increased in mBMSCs from OVX mice compared with SHAM mice. (F) The band intensities of (E) were analyzed by Image J software. GAPDH was used as the internal control. Results are presented as the mean ± SD. (**P < 0.01, compared with SHAM group). [file 13287_2020_1965_MOESM9_ESM.tif]
